# Involvement of plasminogen activator inhibitor-1 and its related molecules in atrial fibrosis in patients with atrial fibrillation

**DOI:** 10.7717/peerj.11488

**Published:** 2021-06-02

**Authors:** Qiaoqiao Li, Yingyu Lai, Xiaoyan Gao, Xin Li, Chun-Yu Deng, Huiming Guo, Junfei Zhao, Hui Yang, Yuwen Xu, Shulin Wu, Yumei Xue, Fang Rao

**Affiliations:** 1 School of Medicine, South China University of Technology, Guangzhou, China; 2 Guangdong Cardiovascular Institute, Guangdong Provincial People’s Hospital, Guangdong Academy of Medical Sciences, Guangzhou, China; 3 Guangdong Provincial Key Laboratory of Clinical Pharmacology, Research Center of Medical Sciences, Guangdong Provincial People’s Hospital, Guangzhou, China

**Keywords:** Bioinformatics analysis, Atrial fibrillation, p53, Plasminogen activator inhibitor-1, Atrial fibrosis

## Abstract

Atrial fibrillation is the most common form of cardiac arrhythmia. Atrial fibrosis is a significant feature of atrial fibrillation though its mechanism is not well understood. We searched the Gene Expression Omnibus database to compare mRNA expression patterns between atrial fibrillation and sinus rhythm samples; one hundred and forty eight differentially expressed genes were identified. Most of these genes were significantly enriched in the extracellular matrix organization process and collagen-activated tyrosine kinase receptor signaling pathway. To screen hub genes involved in atrial fibrosis, we constructed a protein-protein interaction network and found that three hub genes (SERPINE1/plasminogen activator inhibitor-1/PAI-1, TIMP Metallopeptidase Inhibitor 3/TIMP3 and decorin/DCN) play vital roles in atrial fibrosis, especially plasminogen activator inhibitor-1. Elevated plasminogen activator inhibitor-1 expression was positively correlated with the p53 signaling pathway. Plasminogen activator inhibitor-1 and p53 protein expression levels were verified in patients with sinus rhythm and atrial fibrillation by Western blot analysis. Compared with the sinus rhythm controls, p53 and plasminogen activator inhibitor-1 protein expressions were upregulated in the atrial tissues of patients with atrial fibrillation. p53 was also found to regulate plasminogen activator inhibitor-1 based on the results of cellular and molecular experiments. Thus, the p53/plasminogen activator inhibitor-1 signaling axis may participate in the pathophysiological processes of atrial fibrillation, and plasminogen activator inhibitor-1 may serve as a new therapeutic biomarker in atrial fibrillation.

## Introduction

Atrial fibrillation (AF) is the most common form of clinical tachyarrhythmia (Zhang et al., 2019) and can lead to an increased risk of heart failure, stroke, disability, and mortality. A preponderance of evidence has demonstrated that structural and electrical remodeling are involved in the development and maintenance of AF (Avula et al., 2019; Liu et al., 2020; Ozgen et al., 2007). Atrial fibrosis, a significant feature of atrial fibrillation, was considered to result from disturbed extracellular matrix (ECM) metabolism with excessive fibrillar collagen including Col1a1 and Col3a1 deposition, generally in response to a cardiac insult (Hsiao et al., 2016). Currently, the curative treatments of AF contain rate and rhythm control, anticoagulant use and catheter ablation. In particular, catheter ablation is a significant treatment modality for patients with drug-refractory AF. However, the development of atrial substrate that marks AF likely occurs for years before the manifestation of AF onset (Ezeani et al., 2020). Due to the lack of early detection of AF, disability and mortality rates in patients with AF remain high. Thus, there is an urgent demand to identify new biomarkers to develop a better understanding of the molecular mechanisms involved in AF pathogenesis.

In recent decades, large-scale profiling techniques, such as microarray and next-generation sequencing, have been widely applied to screen genetic alterations at the genomic and transcriptomic levels, which has helped identify differentially expressed genes (DEGs) and potential functional pathways involved in the development of AF (Jahid, Huang & Ruan, 2014). In this study, we used bioinformatic methods to analyze original genetic data from the atrial tissue of patients with AF and compared them to those from patients with normal sinus rhythm (SR) to determine an effective biomarker that could become a potential therapeutic target for AF.

## Materials and methods

### Microarray data

The Gene Expression Omnibus (GEO) database (http://www.ncbi.nlm.nih.gov/geo, RRID:CVCL_VR24) is a public repository that provides access to high-throughput genomic datasets (Wang et al., 2019). We downloaded the GSE2240 datasets (Affymetrix GPL 96 platform. Affymetrix Human Genome U133A Array) from the GEO database to use in our analysis. Furthermore, the probes were converted into corresponding gene symbols based on the platform annotation information from the published data. The GSE2240 datasets contained 10 AF tissue samples and 20 SR tissue samples.

### Principal component analyses

All related genes were normalized to their median, and the data quality was assessed by performing a principal component analysis using NetworkAnalyst (https://www.networkanalyst.ca, RRID:SCR_016909) (Tong et al., 2011; Ye et al., 2019).

### Identification of DEGs

DEGs common to the AF and SR samples were identified using NetworkAnalyst. Probe sets without corresponding gene symbols or genes that matched more than one probe set were discarded or averaged, respectively. The absolute value of logFC > 0.5 (FC, fold change) and *P*-value (adjusted) < 0.05 were regarded as statistically significant.

### Gene Ontology and Kyoto Encyclopedia of Genes and Genomes Analysis of DEGs

We performed a Gene Ontology (GO) analysis and a Kyoto Encyclopedia of Genes and Genomes (KEGG) pathway analysis of the DEGs using clusterProfiler package in R (v3.6.0) (Yu et al., 2012). The GO functional categories contained the terms biological process (BP), cellular component (CC), and molecular function (MF). Enrichment significance thresholds were set at *P*-value (adjusted) < 0.05.

### Gene set enrichment analysis of DEGs

We performed a gene set enrichment analysis (GSEA) of the DEGs utilizing the Reactome Pathway Database and the web-based Gene Set Analysis Toolkit version 2.0 (Liao et al., 2019; Wang et al., 2017) (http://bioinfo.vanderbilt.edu/webgestalt/, RRID:SCR_006786). —NES— > 1 and FDR < 0.25 were considered significant (NES, normalized enrichment score; FDR, false discovery rate).

### Gene cluster identification

Next, the candidate DEGs were analyzed using the ClueGO app (Cytoscape; http://apps.cytoscape.org/apps/cluego, RRID:SCR_005748), a functional classification tool that uses the latest precompiled annotation files, including GO and functional pathways enrichment analysis (Bindea et al., 2009; Cao et al., 2018). Significance was defined as a *P* value < 0.05 (Shannon et al., 2003).

### Protein-protein interaction network analysis

Protein-protein interaction (PPI) networks were established according to the STRING analysis (https://string-db.org, RRID:Addgene_36407), and an interacting activity with a combined score >0.4 was deemed statistically significant. Hub modules in the PPI network were then selected using the Molecular Complex Detection (MCODE) plug-in for Cytoscape (Yuan et al., 2018). To screen highly connected hub genes in the PPI network, the Cytoscape cytoHubba plugin was also used (Niemira et al., 2020).

### Identifying differentially expressed mRNAs in the public database

The transcriptional profile data from the highly connected hub genes from the AF and SR samples were downloaded from the GSE2240 datasets. Then, the “ggpubr” R packages was used to measure the mRNA expression level differences between the two groups (AF vs. SR).

### Gene Set Enrichment Analysis

Another GSEA was performed using the GSEA v4.0.3 software (http://software.broadinstitute.org/gsea/index.jsp, RRID:SCR_003199) (Subramanian et al., 2005). We analyzed the SERPINE1/PAI-1 and DCN mRNA level changes in genomic information in GO, BIOCARTA, and KEGG pathways using the GSEA tool. The 30 samples in the GSE2240 datasets (20 SR samples and 10 AF samples) were classified into the low and high expression groups using the SERPINE1 (PAI-1) expression median level as a cut-off point. The potential function of SERPINE1 (PAI-1) (high vs. low) was screened using a GSEA to determine whether defined lists (or sets) of genes exhibited a statistically significant bias in their distribution within a ranked gene list (Tebbji et al., 2014). The expression value of SERPINE1 (high vs. low) was used as the phenotypical label, the number of permutations was set to 1000, and c2.all.v7.1.symbols.gmt from the Molecular Signatures Database (MSigDB) (http://software.broadinstitute.org/gsea/msigdb) was defined as the functional gene set (Liberzon et al., 2015). All other parameters were set to default values. the absolute value of NES > 1 and FDR < 0.25 were chosen as the significance cut-off criteria (D’Haene et al., 2016). The GSEA of DCN was performed using the same method.

### Patients

This investigation was reviewed and approved by the research ethics committee at Guangdong General Hospital, Guangdong Academy of Medical Sciences (Approval No. GDREC20160128H). Left atrial appendages (LAAs) were obtained from SR or AF patients undergoing cardiac thoracotomy. Patients with diabetes, hypertension, pneumonia, or other infectious diseases were excluded from the study. All patients or their legally authorized representative signed the necessary informed consent forms. After surgical excisions were performed, the specimens were immediately snap-frozen in liquid nitrogen and stored at −80 °C before the experiments were performed. Patient data, including age, sex distribution, type of valve disease, and left ventricular function are shown in Table S1.

### Isolation and culture of human atrial fibroblasts

Human atrial fibroblasts were isolated from the LAAs using direct adherent culture methods. In brief, the tissue specimens (approximately 200 mg) were cut into approximately 1 mm^3^ pieces and suspended in 500 µL of fetal bovine serum and then placed in T-25 flasks. Two hours later, the flasks were flooded with 5 mL of complete media (Fibroblast Basal Medium [Lonza, CC-3131], 10% fetal bovine serum [Gibco], Insulin [Lonza, CC-4021WW], rhFGF [Lonza, CC-4065WW], GA-1000 [Lonza, CC-4081WW]). The tissues were cultured in a humidified incubator at 37 °C and 5% CO_2_. After approximately 5 days, new cells migrated from the edge of the tissues and when they reached 80% confluence, cells were passaged. Cells were plated at densities of 80,000 cells/well in 6-well plates for the p53 knockdown experiment.

A mixture of three p53 siRNA chain (hTP53 si-1 sense GCG CAC AGA GGA AGA GAA UTT; hTP53 si-2 sense CCA CUG GAU GGA GAA UAU UTT; hTP53 si-3 sense CCA UCC ACU ACA ACU ACA UTT; Jikai Biotechnology Co. Shanghai, China) was used to knock down protein expression of p53. When the cells reached 70–80% confluence, transfection was performed according to the following method. First, 6 µL of Lipofectamine 3,000 Reagent (Thermo Fisher) and 5 µL p53 siRNA/well (final concentration: 50 nM) were diluted in 250 µL of Opti-MEM reduced serum medium (Gibco) respectively, and then mixed them well. After incubation for 10–15 min at room temperature, the complexes were added directly to cells in 1.5 mL fresh complete culture medium. The cells were then continuously cultured for 48 h. Finally, they were harvested, and protein expression was detected by Western blot.

### Western blot analysis

Detailed experiment procedure was as previously reported method (Li et al., 2020). Antibodies used for these experiments are listed as follows: (anti-Collagen I antibody [1:2000, Abcam Cat^#^ ab34710, RRID:AB_731684]; anti-Collagen III antibody [1:2000, Abcam Cat^#^ ab7778, RRID:AB_306066]; anti-p53 antibody [1:1000, Cell Signaling Technology Cat^#^ 2524, RRID:AB_331743]; anti-PAI-1 antibody [1:1000, Cell Signaling Technology Cat^#^ 11907, RRID:AB_2797763]; anti-p21 antibody [1:1000, Proteintech 27296-1-AP, RRID:AB_2880834]; anti-TGF-*β* antibody [1:1000, Cell Signaling Technology Cat^#^ 3711, RRID:AB_2063354]. Anti-GAPDH antibody [1:5000, Proteintech Cat^#^ 60004-1-lg, RRID:AB_2107436].

### Statistical analysis

All values are expressed as means ± standard error of mean (SEM), and Student’s *t*-test was used to determine pairwise statistical significance of the differences between two group means. One-way analysis of variance was used for comparisons of multiple groups. A *P*-value < 0.05 was considered statistically significant.

## Results

### Identification of DE-mRNAs

**Figure 1:**
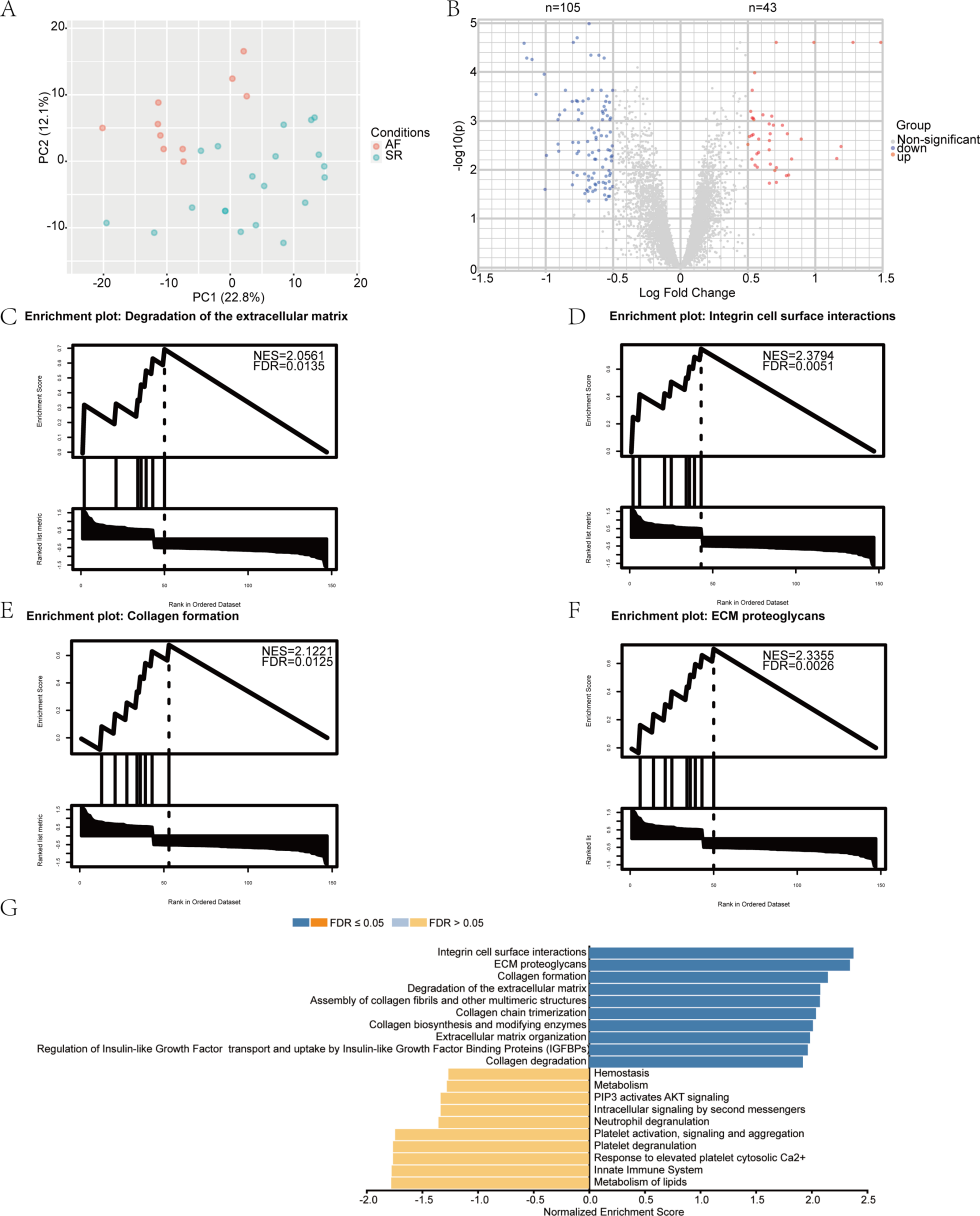
Identification of differentially expressed mRNAs and gene set enrichment analysis (GSEA) results. (A) The principal component analysis (PCA) and sample location. The red dot represents atrial fibrillation (AF) samples, and the green dot represents sinus rhythm (SR) samples. (B) The volcano plot of differentially expressed mRNAs in the GSE2240 dataset. The red dot represents upregulated mRNAs, and the blue dot represents downregulated mRNAs. GSEA results. (C–F) The top four most significantly enriched Reactome terms; (G) The top 10 terms of the GSEA analysis result of differentially expressed genes (DEGs), including positively and negatively enriched results.

We performed a principal component analysis (PCA) of the samples (Fig. 1A), and the results showed good separation of AF from the normal samples. Pre- and post-normalization of data was described in Fig. S1A. Furthermore, —log_2_ FC— > 0.5 and *p*-value (adjusted) < 0.05 were considered as the criteria to identify the DEGs. A total of 148 differentially expressed mRNAs were selected from the 30 samples, among which 105 genes were downregulated and 43 were upregulated in the AF group. The distribution of the DEGs is represented by a volcano map (Fig. 1B). DEGs were ordered according to the *p*-value (adjusted) and the top 50 genes were selected for plotting in heatmap (Fig. S1B). The 148 genes showing differential expression are listed in Table 2.

**Figure 2:**
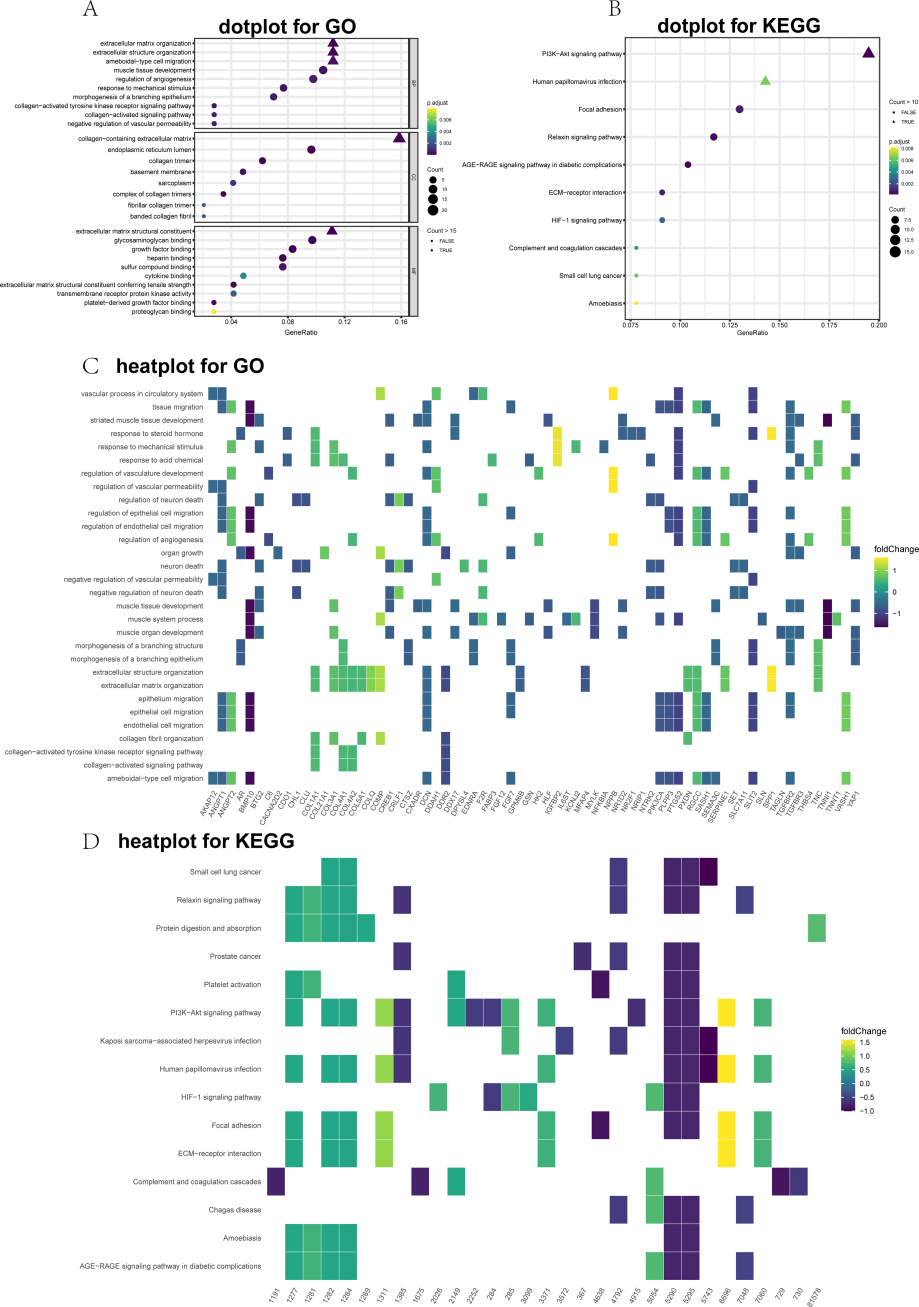
Top 10 significantly enriched Gene Ontology (GO) and Kyoto Encyclopedia of Genes and Genomes (KEGG) terms for differentially expressed genes (DEGs). (A) GO dot plot; (B) KEGG dot plot; (C) GO heat plot; and (D) KEGG heat plot.

### GSEA analysis of the DEGs

To gain further insight into the biological functions of the DEGs, we performed GSEA using the WebGestalt Gene Set Analysis Toolkit, version 2.0. The four most significantly enriched Reactome terms are illustrated in Figs. 1C–1F, including positive enrichment in the integrin cell surface interaction, degradation of the extracellular matrix, collagen formation, and extracellular matrix (ECM) proteoglycans. The top 10 GSEA terms are displayed as a bar chart in Fig. 1G.

### Enrichment analysis of the DEGs

GO and KEGG annotations were performed utilizing the clusterProfiler package for R (v3.6.0). The DEGs were divided into three main groups: CCs, MFs, and BPs. The top 10 GO items were significantly enriched (Fig. 2A). The GO term analysis showed that the ECM organizational process was the most significant enrichment in BPs, followed by the extracellular structure organizational process. Under the CC, the DEGs were significantly enriched for the collagen-containing ECM, endoplasmic reticulum lumen, and collagen trimer. In the MF category, the most abundant term was the ECM structural constituent. To further visualize these results, we generated heat maps to depict fold changes in the expression levels of genes associated with several GO terms, including ECM organization (Fig. 2C). Dot plots of the enriched KEGG pathways are shown in Fig. 2B. The PI3K-Akt signaling pathway, human papillomavirus infection, and focal adhesion were significantly enriched. Furthermore, the heatmap showed DEGs related to the enriched signaling pathways (Fig. 2D).

### ClueGO analysis

**Figure 3:**
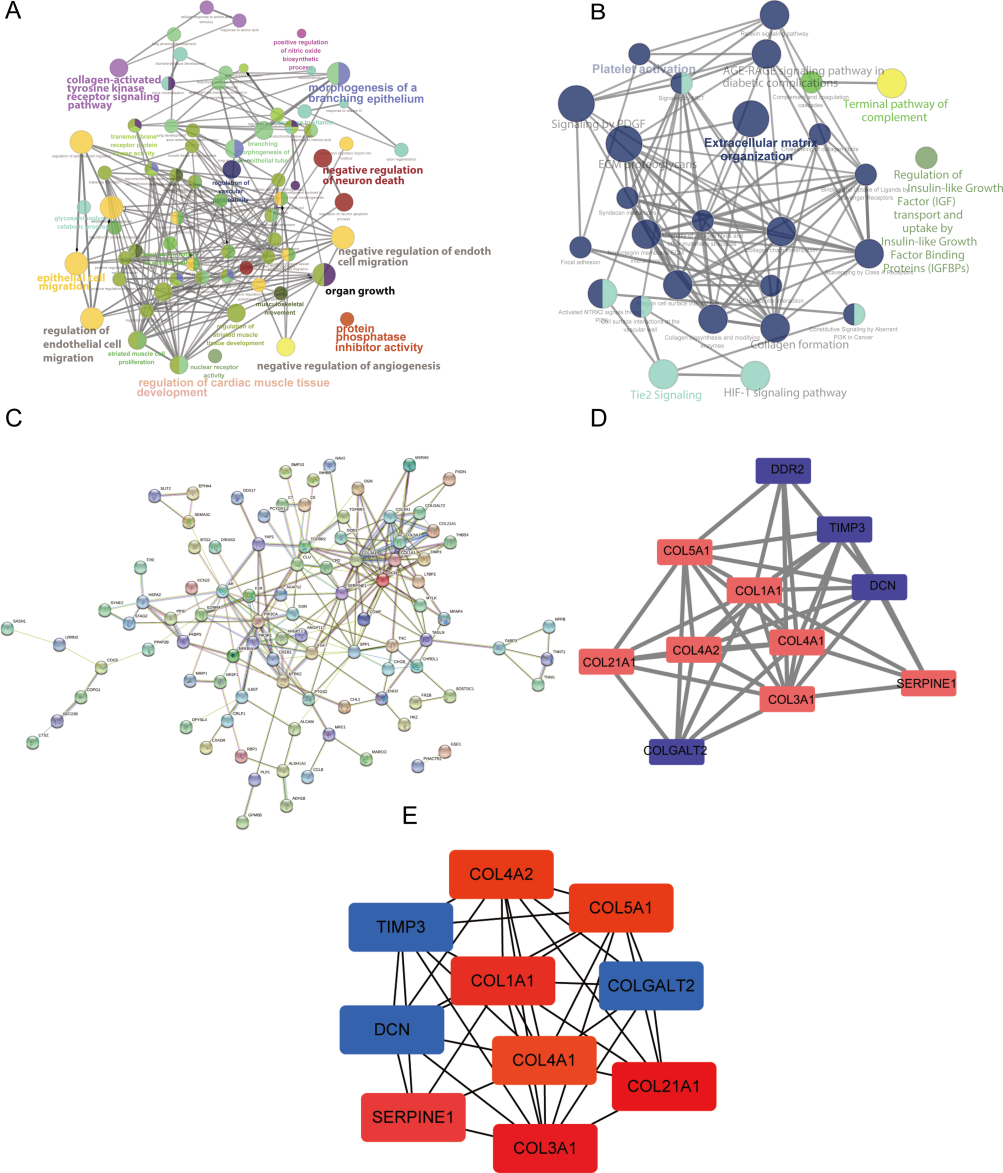
Analysis using ClueGO and CluePedia apps in Cytoscape (version 3.7.2) and protein-protein interaction (PPI) networks of the differentially expressed genes (DEGs). (A) The significantly enriched Gene Ontology (GO) terms of the DEGs were identified. (B) The results of the pathway enrichment analysis. Circle size represents the degree of enrichment. (C) The PPI network. (D and E) The hub modules and genes, red and green indicate high and low expressions, respectively.

GO, KEGG, and Reactome analyses were performed using ClueGO and CluePedia in the Cytoscape software to explore the key functions of the target genes. The ClueGO analysis indicated that the collagen-activated tyrosine kinase receptor signaling pathway, regulation of endothelial cell migration, regulation of cardiac muscle tissue development, and organ growth were significantly enriched GO terms (Fig. 3A). Furthermore, the results of our pathway enrichment analysis showed significant associations between ECM organization, HIF-1 signaling pathway, Tie2 signaling pathway, platelet activation and signaling by PDGF (platelet derived growth factor) pathway, and the 148 DEGs (Fig. 3B).

### PPI network analysis

We used Cytoscape (https://cytoscape.org/) (version 3.7.2) to perform a visual analysis of the protein-protein association networks for predicting the interactions of the proteins encoded by the DEGs (Yu et al., 2020). The PPI network was constructed as shown in Fig. 3C. The hub modules of the PPI network were selected using MCODE. The most significant module was screened out with a cutoff MCODE score of > 5 (Fig. 3D). Furthermore, the top 10 hub genes were identified using cytoHubba (Fig. 3E). SERPINE1, DCN, and TIMP3 were included in this analysis.

### GO analysis of the hub genes and the relative mRNA expression of the three hub genes in the public database

**Figure 4:**
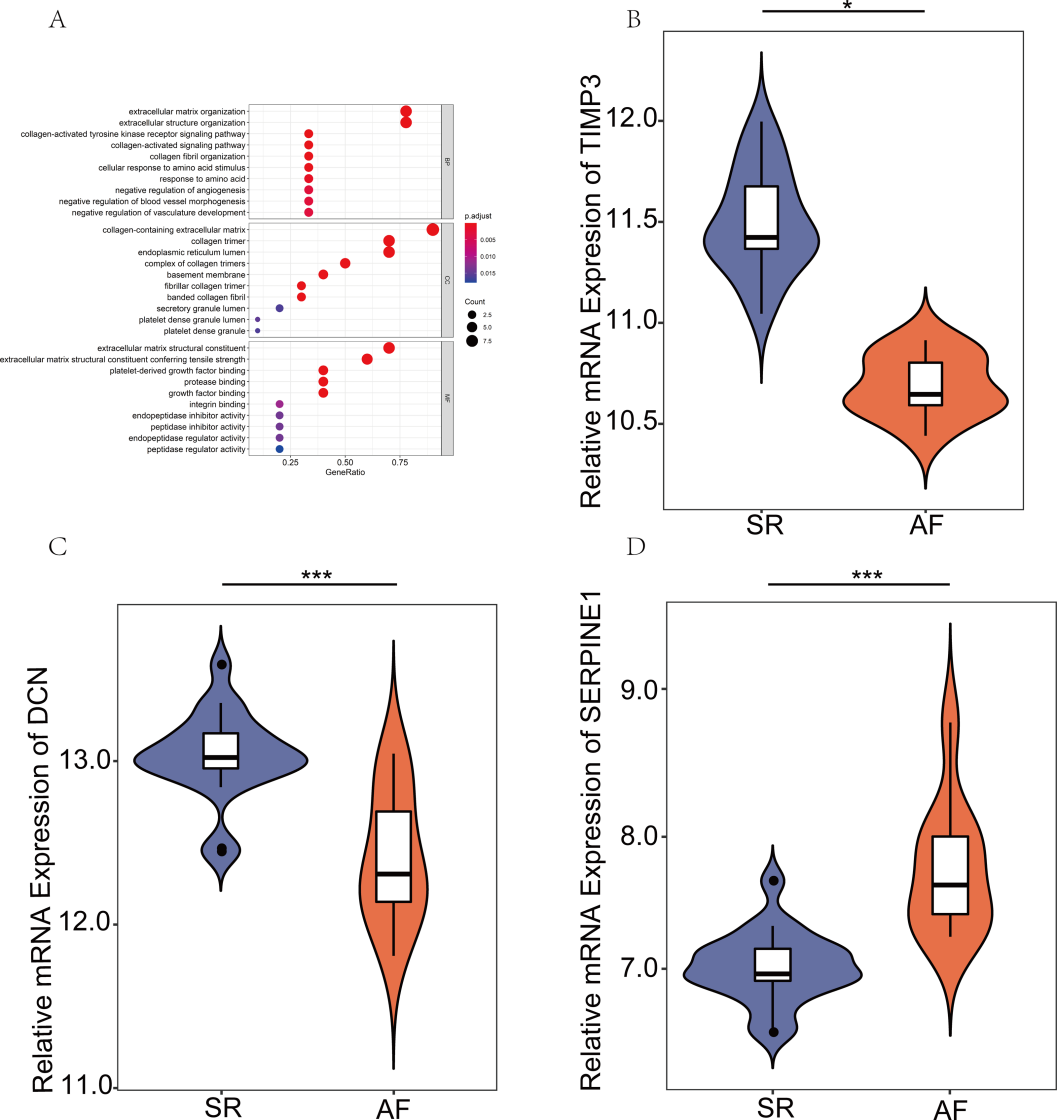
Expression verification of key genes in the public database. (A) Gene Ontology analyses were employed to perform the functional enrichment analysis of key genes. (B–D) Differentially expressed genes (DEGs) were visualized using R version 3.6.3 in GSE2240. (*P* values: *, *P* < 0.05; **, *P* < 0.01; ***, *P* < 0.001. sinus rhythm (SR) vs. atrial fibrillation (AF)).

To systematically assess the potential biological functions of the candidate genes, we performed GO enrichment analysis using clusterProfiler for R 3.6.0. The GO analysis indicated that these hub genes were significantly associated with the term ECM organizational process (Fig. 4A). Moreover, the three hub genes, SERPINE1 (PAI-1), DCN, and TIMP3, were differentially expressed. SERPINE1 was highly expressed in AF, and the two other genes exhibited opposite expression levels (Figs. 4B–4D).

### GSEA associated with SERPINE1(PAI-1) and DCN expression

**Figure 5:**
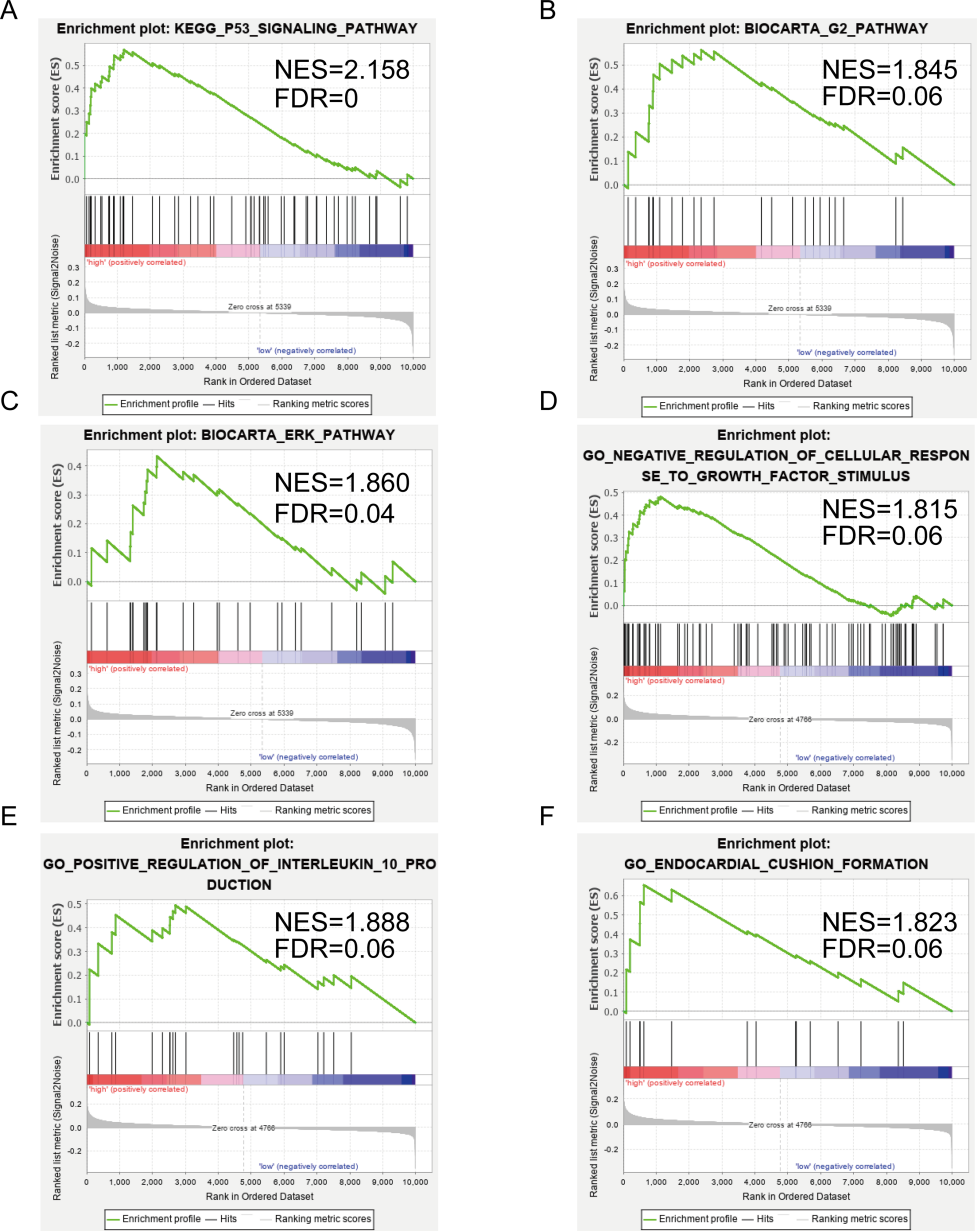
Gene set enrichment analysis (GSEA) enriched Gene Ontology, Kyoto Encyclopedia of Genes and Genomes (KEGG), and BIOCART pathways in the GSE2240 dataset of SERPINE1 (PAI-1) and decorin (DCN) expression. (A–C) SERPINE1. (D–F) DCN.

Figures 5A–5C show the significantly enriched KEGG pathways of SERPINE1 (PAI-1): p53 signaling pathway, G2 pathway, and ERK pathway. Figures 5D–5F show the main enriched GO of DCN: negative regulation of cellular response to growth factor stimulus, positive regulation of interleukin 10 production, and endocardial cushion formation.

### PAI-1 was highly expressed in AF patients

**Figure 6:**
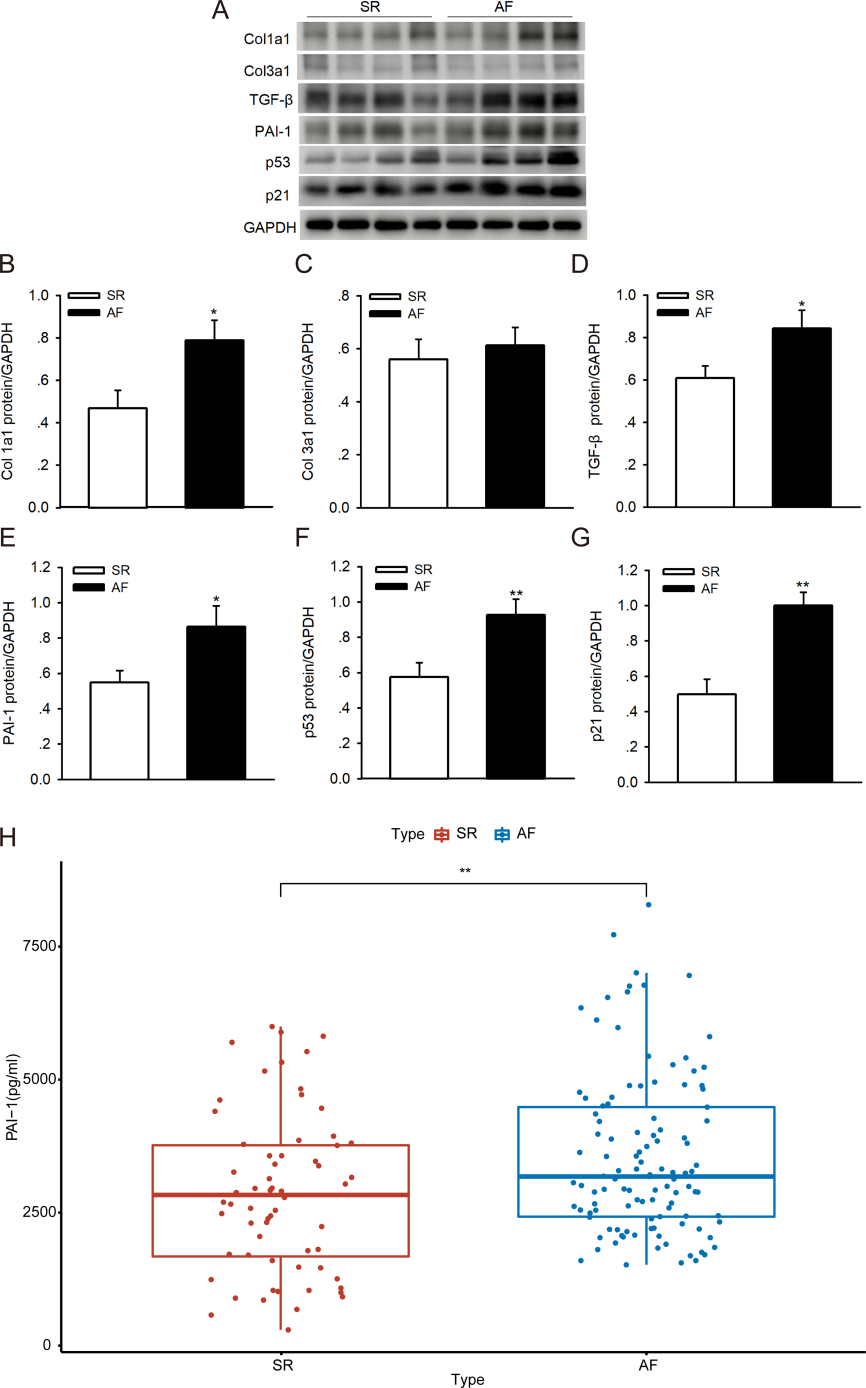
PAI-1 and p53 levels in patients with sinus rhythm (SR) and atrial fibrillation (AF). (A–G) Representative blots and densitometry analysis of PAI-1, p53, p21, Col1a1/3a1 and TGF-*β* proteins in left atrial appendages (LAA) tissues in patients with SR (*n* = 16) and AF (*n* = 16). Glyceraldehyde 3-phosphate dehydrogenase (GAPDH) served as an internal control. (H) Serum levels of PAI-1 were higher in patients with AF. *P < 0.05, **P < 0.01 vs. SR.

To investigate whether p53/PAI-1 plays a vital role in the pathogenesis of AF, we addressed the PAI-1, p53, p21, Col1a1/3a1 and TGF-*β* protein expression levels in LAAs from patients with AF and the SR controls. We found that the protein levels of PAI-1, p53, p21, Col1a1/3a1 and TGF-*β* were higher in the AF samples than in the SR controls (Figs. 6A–6G). The PAI-1 serum levels were also significantly higher in patients with AF (Fig. 6H) (the patient characteristics are shown in Table S3). These results indicate that p53/PAI-1 might be involved in the pathophysiology of atrial fibrosis.

### Knocking down p53 reduces the expression level of PAI-1 in human atrial fibroblasts

**Figure 7:**
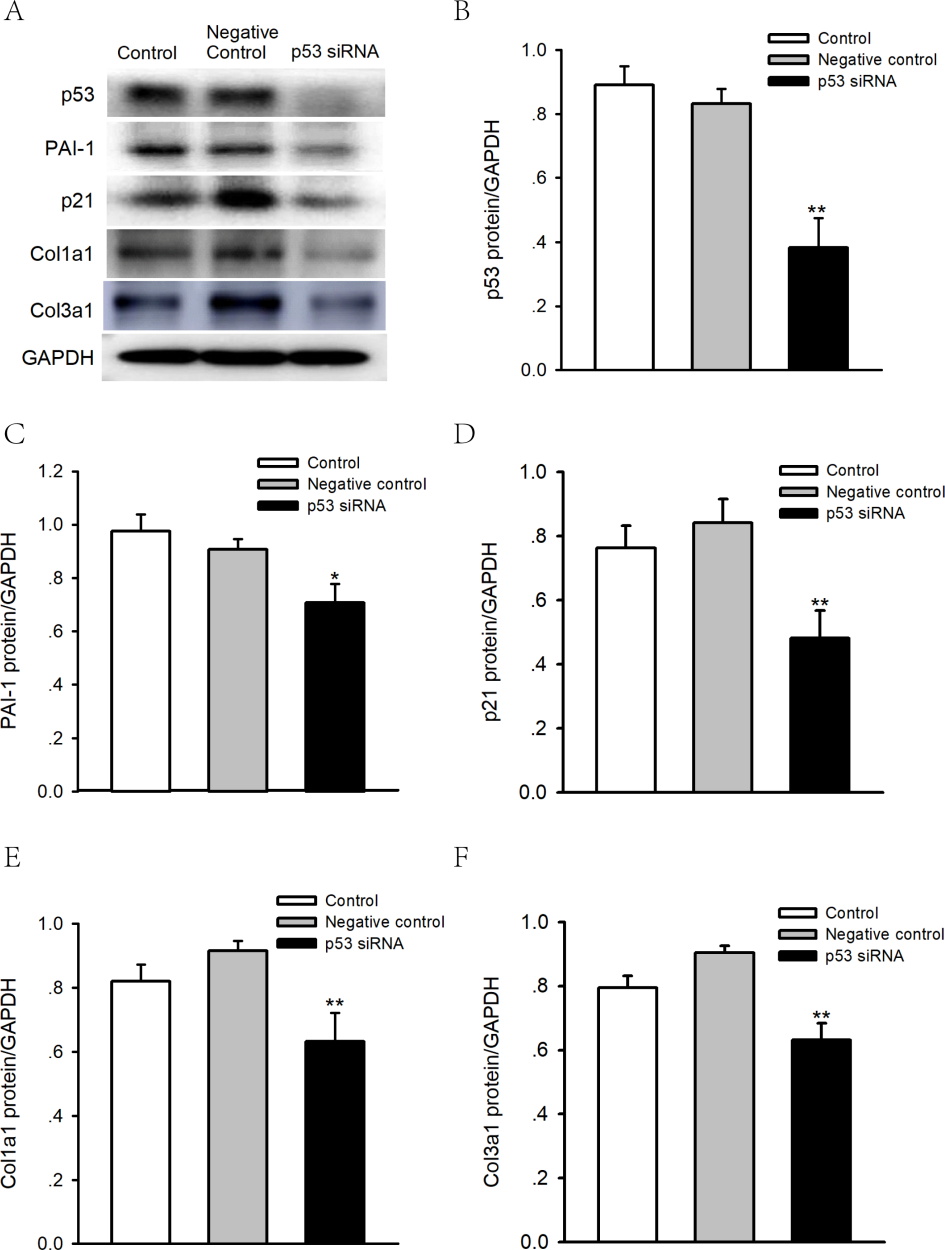
Effects of p53 knockdown on protein expression levels of PAI-1 and Col 1A1/3A1 in human atrial fibroblasts. (A–F) Representative blots and densitometry analysis of PAI-1, p21 and Col 1a1/3a1 protein expression with or without p53 knockdown in human atrial fibroblasts. Glyceraldehyde 3-phosphate dehydrogenase (GAPDH) was used as an internal control. Scramble siRNA was used as negative control. *P < 0.05, **P < 0.01 vs. Negative control.

To determine whether p53 is involved in the regulation of PAI-1, we performed p53 knockdown using siRNA in human atrial fibroblasts. As shown in Figs. 7A–7F, knocking down p53 significantly inhibited PAI-1, p21 and Col 1a1/3a1 protein expression. As such, p53 might regulate the expression of PAI-1 and fibrosis in human atrial fibroblasts, thus leading to the structural remodeling of the atrium and, therefore, AF.

## Discussion

The incidence of AF is associated with structural, electrical, genomic, hormonal, and autonomic atrial remodeling (Lv et al., 2019). Development and progression of atrial fibrosis is the hallmark of structural arrhythmogenic remodeling in AF and is considered the substrate for AF occurrence, but the specific molecular mechanism of atrial fibrosis is still unclear. Currently, the microarray technology can help to screen genetic alterations associated with the disease, and it has been proven to be essential for identifying new biomarkers.

In the present study, the GSE2240 mRNA dataset was analyzed to identify DEGs common to atrial tissues from AF and SR patients. In total, 148 DEGs, including 43 upregulated genes and 105 downregulated genes, were screened in the AF samples. Furthermore, to obtain further insight into the biological roles of the DEGs, we performed GO, KEGG, and Reactome enrichment analyses and found that these genes are mainly involved in the organizational process of the extracellular structures, collagen formation, and ECM proteoglycan signaling pathway. Based on these results, all the DEGs analyzed in this study could be associated with structural remodeling. To test this hypothesis, we used the ClueGO and CluePedia apps in Cytoscape 3.7.2 to explore the functions of these target genes. The results indicate that the terms collagen-activated tyrosine kinase receptor signaling pathway, regulation of endothelial cell migration, regulation of cardiac muscle tissue development, ECM organization, HIF-1 signaling pathway, and Tie2 signaling pathway were significantly enriched. Next, to search for hub genes involved in structural remodeling, we constructed PPI networks of the DEGs. MCODE and cytoHubba were used to identify the hub modules and hub genes. Our results suggest that SERPINE1 (PAI-1), TIMP3, and DCN are promising candidates for further analysis.

In addition, we undertook GO analysis to perform functional enrichment of key genes, including SERPINE1(PAI-1), TIMP3, and DCN. Similarly, the terms significant enrichment of ECM organization, collagen-containing ECM, and ECM structural constituent were found in this analysis. We next validated the relative mRNA expression of SERPINE1, TIMP3, and DCN in the GSE2240 dataset (AF vs SR). The results show that SERPINE1 is highly expressed in AF. However, TIMP3 and DCN are expressed at low levels in AF.

TIMP3, as a member of the TIMP family, can regulate many physiological effects, including cell growth, hypertrophy, migration, and cardiac fibrosis, through a matrix metalloproteinase (MMP)-dependent or -independent manner (Zhang et al., 2018). It has also been reported that TIMP3 levels are significantly reduced in patients with dilated cardiomyopathy and heart failure, and even the loss of a single TIMP3 allele in mice leads to myocardial fibrosis. Thus, TIMP3 can be a powerful therapeutic candidate for strategies aimed at blocking myocardial fibrosis in the early stages of heart disease (Kassiri et al., 2009).

DCN is a leucine-rich proteoglycan constituent of the ECM and has powerful antifibrotic, antioxidant, anti-inflammation, and antiangiogenic properties in cardiovascular diseases (Thu Thi et al., 2018). Several studies have demonstrated its efficiency in targeting DCN to inhibit TGF-*β* availability (Daum et al., 2020; Thu Thi et al., 2018). These results indicate that DCN may be a protective factor against AF.

Finally, we performed a GSEA to further explore the possible mechanisms of SERPINE1 and DCN action. The elevated DCN expression was unexpectedly found to be positively associated with a negative regulation of the cellular response to growth factor stimuli. This result further supports the assertion that DCN exerts an inhibitory influence against cardiac fibrosis. Additionally, elevated SERPINE1(PAI-1) expression is positively correlated with the p53 signaling, G2, and ERK pathways. All these data could direct efforts to confirm the role of the p53/PAI-1 signaling axis in AF.

Plasminogen activator inhibitor-1 (PAI-1), coded by SERPINE1, is a procoagulant factor, promoting the deposition of fibrin and platelet activation, secretion and aggregation. Previous studies have found that plasma levels of PAI-1 were increased in patients with AF and thrombus (Cheng et al., 2019). After catheter ablation, it then decreased significantly (Otto et al., 2018). Increased circulating PAI-1 was also significantly associated with subsequent stroke in patients with AF (Wu et al., 2015). However, recent studies have indicated that PAI-1 was related to the progression of AF (DeWith et al., 2020). It also reported that PAI-1 can be as a predictor of postoperative AF after cardiopulmonary bypass. This suggested the hypothesis that drugs that decrease PAI-1 antigen could influence the risk of AF (Pretorius et al., 2007). To sum up, PAI-1 was closely related to the occurrence and prognosis of AF. However, the mechanisms responsible for PAI-1’s involvement in AF are not clear.

In our study, we identified DCN, TIMP3 and PAI-1 as the hub genes in the process of atrial fibrosis in AF. More importantly, we found that p53, PAI-1, and the cardiac fibrosis biomarker showed increased expression in atrial tissue from AF patients. The serum levels of PAI-1 were also significantly higher in patients with AF. In addition, inhibition of p53 expression can suppress expression of PAI-1 and the cardiac fibrosis biomarker in human atrial fibroblasts, which indicated that p53/PAI-1 signal axis may promote the pathological process of atrial fibrosis and then modified the atrial substrate and potentially lead to AF. Our study provides new evidence and ideas for further exploration of the mechanism and treatment of AF.

There are also several limitations still detected in our present study. Firstly, the number of samples we obtained from GSE2240 was small, which can cause bias when analyzing the DE-mRNAs. Thus, further research with larger sample sizes is needed for validation. Secondly, the functions and molecular mechanisms of genes are quite complicated, and predictions based only on bioinformatics need cellular, animal experiments and clinical trials for further verification.

## Conclusion

In this study, we searched the Gene Expression Omnibus (GEO) database to compare mRNA expression between AF and sinus rhythm (SR) samples, and in doing so, we identified 148 differentially expressed genes. To screen out hub genes involved in atrial fibrosis, we constructed a protein-protein interaction (PPI) network. We found that three hub genes (SERPINE1, TIMP3 and DCN) play vital roles in atrial fibrosis, particularly SERPINE1 (PAI-1). Elevated SERPINE1 (PAI-1) expression was also positively correlated with the p53 signaling pathway. We believe that our study makes a significant contribution to the literature because our results suggest that the p53/PAI-1 signaling axis may participate in AF and SERPINE1 (PAI-1) may serve as a new therapeutic AF biomarker.

##  Supplemental Information

10.7717/peerj.11488/supp-1 Supplemental Information 1 Analysis of data quality control and heat map plot of DEGs The pre-normalized data vs post-normalized data (Figure S1A) were plotted. Heat maps of the differentially expressed genes are in Figure S1B.

10.7717/peerj.11488/supp-2 Supplemental Information 2 Baseline characteristics of patients N, number of patients; EF, ejection fraction; AVR, aortic valve replacement; MVR, mitral valve replacement; SR, sinus rhythm; AF, atrial fibrillation. * P < .05 vs. SR, ** P < .01 vs. SR. Values are mean ±SEM. Student’s *t*-test or Chi-square test was used to evaluate differences between two groups.

10.7717/peerj.11488/supp-3 Supplemental Information 3 The details of differentially expressed genes (DEG)

10.7717/peerj.11488/supp-4 Supplemental Information 4 GSEA raw data Raw data exported from the GSEA software applied for data analyses and preparation for Fig. 5.

10.7717/peerj.11488/supp-5 Supplemental Information 5 Baseline characteristics of patients N, number of patients; EF, ejection fraction; CCB, calcium channel blockers; SR, sinus rhythm; AF, atrial fibrillation. *P ¡ .05 vs. SR, **P ¡ .01 vs. SR. Values are presented as mean±SEM. Student’s *t*-test or Mann–Whitney U-test was used to evaluate differences between two groups.

10.7717/peerj.11488/supp-6 Supplemental Information 6 Plasma PAI-1 levels in patients with sinus rhythm and atrial fibrillation

10.7717/peerj.11488/supp-7 Supplemental Information 7 MCODE network clustering analysis Raw data exported from the Cytoscape software applied for data analyses and preparation for Figs. 3D–3E.

10.7717/peerj.11488/supp-8 Supplemental Information 8 Raw data downloaded from GSE2240 performed for data analyses for Figs. 1A–1B

10.7717/peerj.11488/supp-9 Supplemental Information 9 Raw Western Blot Images

## Additional Information and Declarations

### Data Availability

The following information was supplied regarding data availability: The raw measurements are available in the Supplemental Files.
