# Supplementary figures and images for: Involvement of plasminogen activator inhibitor-1 and its related molecules in atrial fibrosis in patients with atrial fibrillation
Source: PeerJ. 2021 Jun 2;9:e11488. doi: 10.7717/peerj.11488 (PMC8179226; doi:10.7717/peerj.11488)

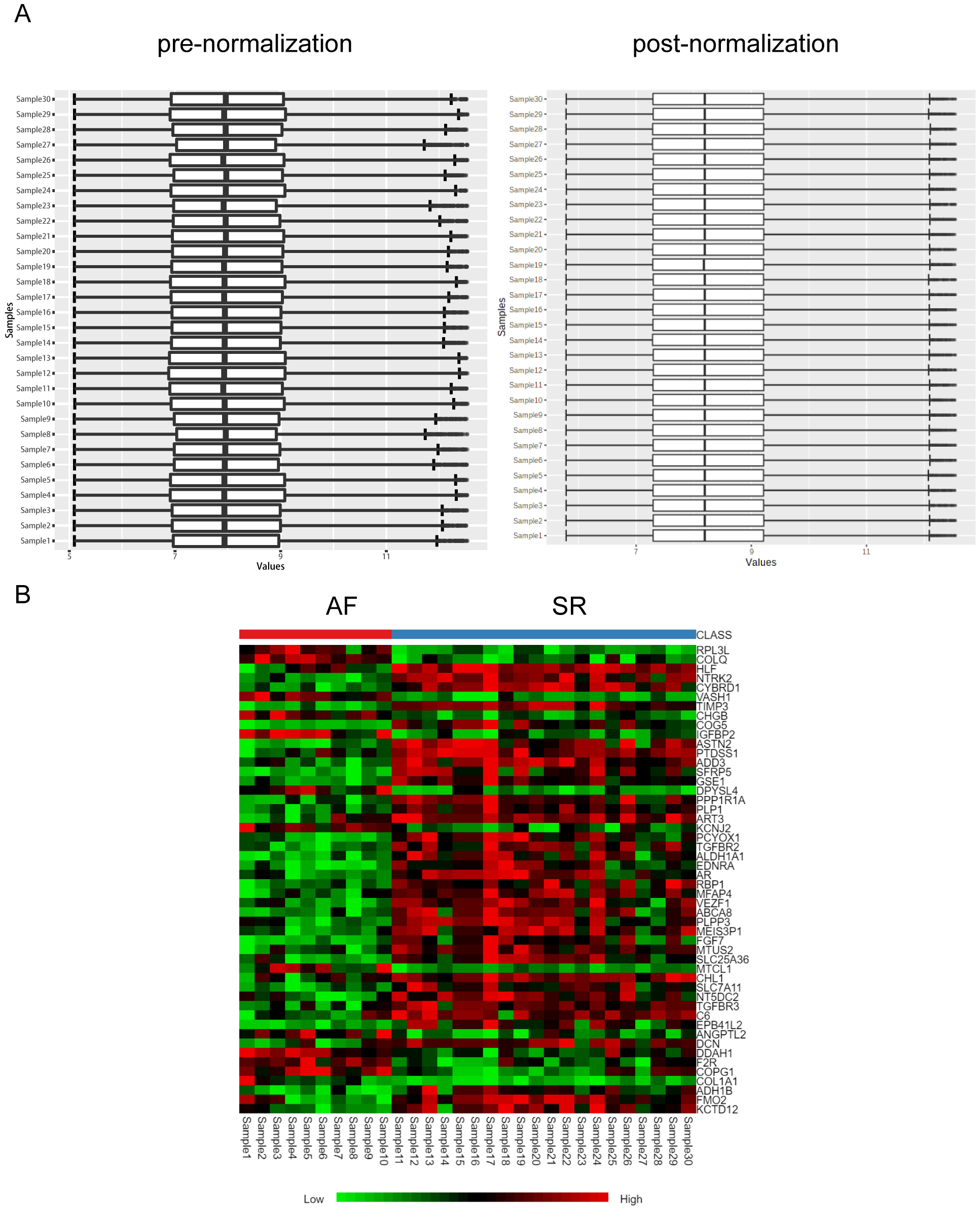

Supplement: Supplemental Information 1 — The pre-normalized data vs post-normalized data (Figure S1A) were plotted. Heat maps of the differentially expressed genes are in Figure S1B. [file peerj-09-11488-s001.png]

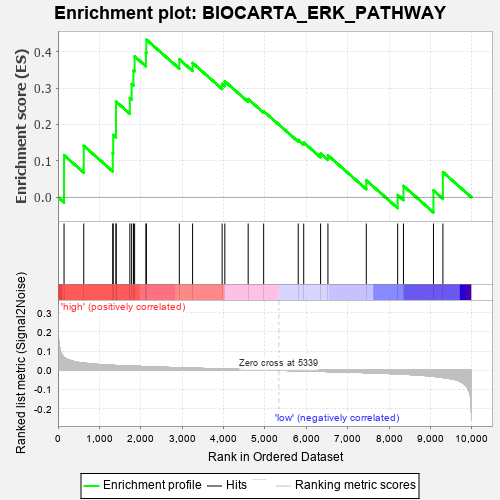

Supplement: Supplemental Information 4 — Raw data exported from the GSEA software applied for data analyses and preparation for Fig. 5. [file peerj-09-11488-s004.zip › GSEA_RAW_DATA/enplot_BIOCARTA_ERK_PATHWAY_21.png]

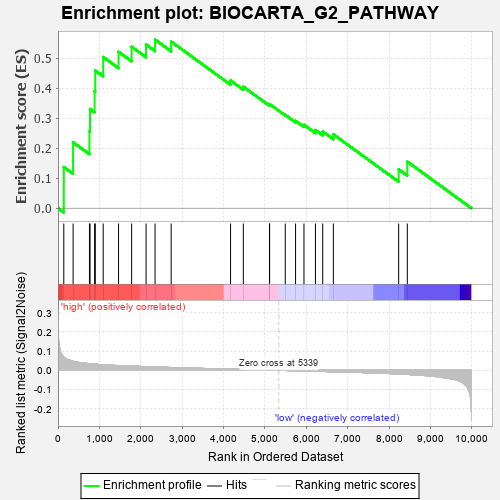

Supplement: Supplemental Information 4 — Raw data exported from the GSEA software applied for data analyses and preparation for Fig. 5. [file peerj-09-11488-s004.zip › GSEA_RAW_DATA/enplot_BIOCARTA_G2_PATHWAY_27.png]

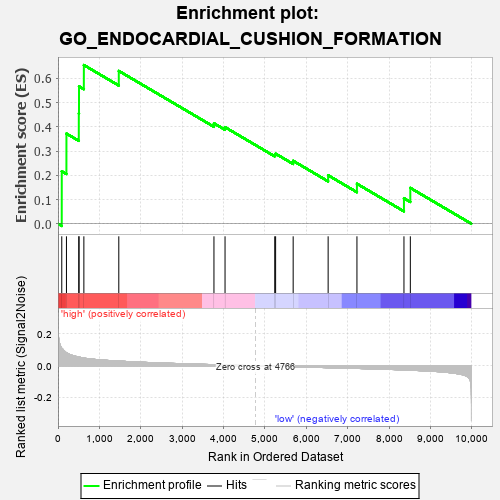

Supplement: Supplemental Information 4 — Raw data exported from the GSEA software applied for data analyses and preparation for Fig. 5. [file peerj-09-11488-s004.zip › GSEA_RAW_DATA/enplot_GO_ENDOCARDIAL_CUSHION_FORMATION_164.png]

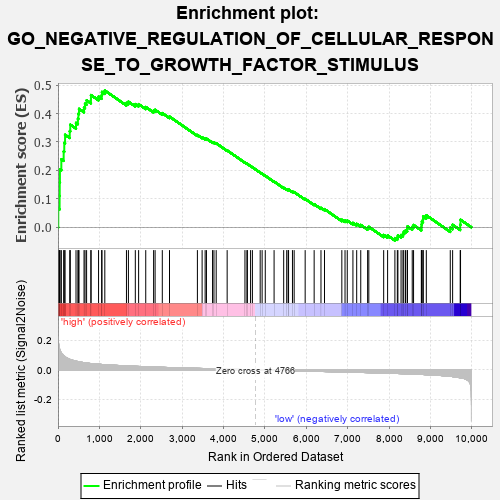

Supplement: Supplemental Information 4 — Raw data exported from the GSEA software applied for data analyses and preparation for Fig. 5. [file peerj-09-11488-s004.zip › GSEA_RAW_DATA/enplot_GO_NEGATIVE_REGULATION_OF_CELLULAR_RESPONSE_TO_GROWTH_FACTOR_STIMULUS_173.png]

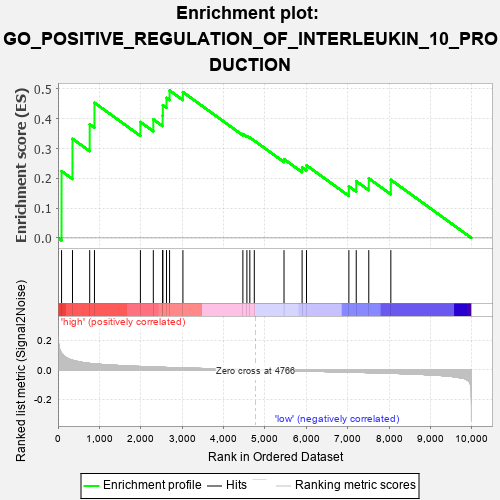

Supplement: Supplemental Information 4 — Raw data exported from the GSEA software applied for data analyses and preparation for Fig. 5. [file peerj-09-11488-s004.zip › GSEA_RAW_DATA/enplot_GO_POSITIVE_REGULATION_OF_INTERLEUKIN_10_PRODUCTION_146.png]

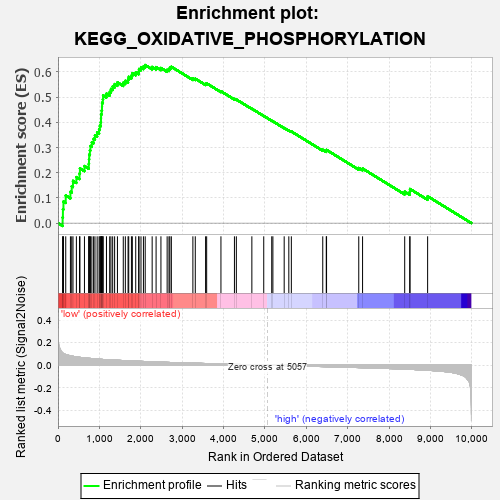

Supplement: Supplemental Information 4 — Raw data exported from the GSEA software applied for data analyses and preparation for Fig. 5. [file peerj-09-11488-s004.zip › GSEA_RAW_DATA/enplot_KEGG_OXIDATIVE_PHOSPHORYLATION_24.png]

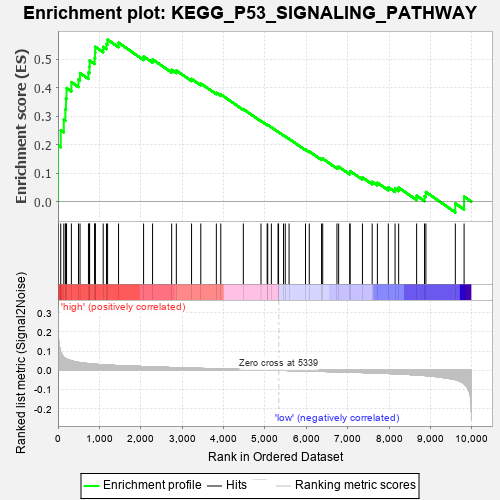

Supplement: Supplemental Information 4 — Raw data exported from the GSEA software applied for data analyses and preparation for Fig. 5. [file peerj-09-11488-s004.zip › GSEA_RAW_DATA/enplot_KEGG_P53_SIGNALING_PATHWAY_3.png]

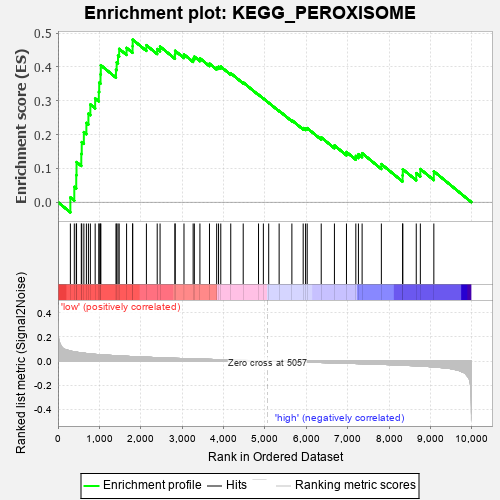

Supplement: Supplemental Information 4 — Raw data exported from the GSEA software applied for data analyses and preparation for Fig. 5. [file peerj-09-11488-s004.zip › GSEA_RAW_DATA/enplot_KEGG_PEROXISOME_60.png]

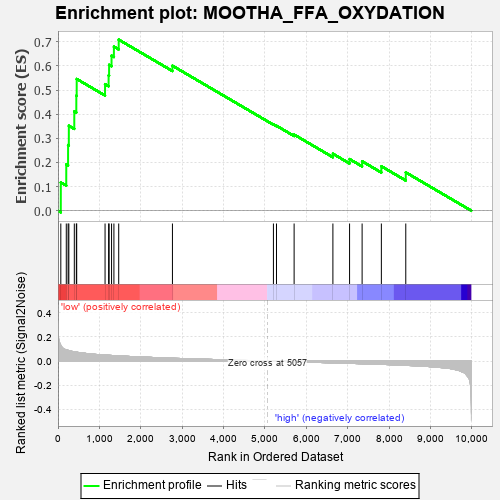

Supplement: Supplemental Information 4 — Raw data exported from the GSEA software applied for data analyses and preparation for Fig. 5. [file peerj-09-11488-s004.zip › GSEA_RAW_DATA/enplot_MOOTHA_FFA_OXYDATION_9.png]

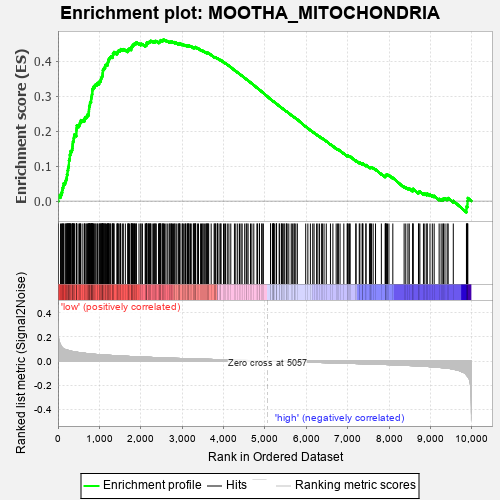

Supplement: Supplemental Information 4 — Raw data exported from the GSEA software applied for data analyses and preparation for Fig. 5. [file peerj-09-11488-s004.zip › GSEA_RAW_DATA/enplot_MOOTHA_MITOCHONDRIA_18.png]

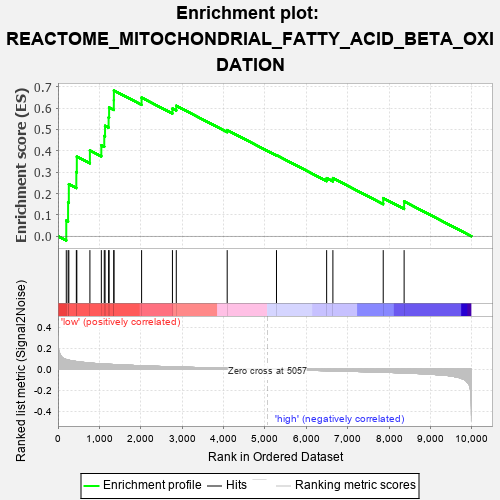

Supplement: Supplemental Information 4 — Raw data exported from the GSEA software applied for data analyses and preparation for Fig. 5. [file peerj-09-11488-s004.zip › GSEA_RAW_DATA/enplot_REACTOME_MITOCHONDRIAL_FATTY_ACID_BETA_OXIDATION_3.png]
