# Supplementary material for: Involvement of plasminogen activator inhibitor-1 and its related molecules in atrial fibrosis in patients with atrial fibrillation
Source: PeerJ. 2021 Jun 2;9:e11488. doi: 10.7717/peerj.11488 (PMC8179226; doi:10.7717/peerj.11488)
Supplement: Supplemental Information 2 — N, number of patients; EF, ejection fraction; AVR, aortic valve replacement; MVR, mitral valve replacement; SR, sinus rhythm; AF, atrial fibrillation. * P < .05 vs. SR, ** P < .01 vs. SR. Values are mean ±SEM. Student’s t-test or Chi-square test was used to evaluate differences between two groups. [file peerj-09-11488-s002.docx]

Supplementary Table 1.

Baseline characteristics of patients.

|  | SR | AF |
| --- | --- | --- |
| N | 16 | 16 |
| men(n) | 6 | 5 |
| women(n) | 10 | 11 |
| Age(y) | 45.38±15.79 | 46.38±12.43 |
| EF (%) | 64.44±6.51 | 62.44±6.97 |
| AVR (n) | 3 | 4 |
| MVR (n) | 5 | 10 |
| Beta Blocker (n) | 3 | 1 |
| Diuretics (n) | 10 | 13 |
| Digoxin (n) | 7 | 7 |

N, number of patients; EF, ejection fraction; AVR, aortic valve replacement; MVR, mitral valve replacement; SR, sinus rhythm; AF, atrial fibrillation. ^⁎^P < .05 vs. SR, ^⁎⁎^P < .01 vs. SR. Values are mean ± SEM. Student's t-test or Chi-square test was used to evaluate differences between two groups.
