# Supplementary material for: Involvement of plasminogen activator inhibitor-1 and its related molecules in atrial fibrosis in patients with atrial fibrillation
Source: PeerJ. 2021 Jun 2;9:e11488. doi: 10.7717/peerj.11488 (PMC8179226; doi:10.7717/peerj.11488)
Supplement: Supplemental Information 3 [file peerj-09-11488-s003.docx]

Supplementary Table 2

The DEGs

| Up/Down | Count | Genes |
| --- | --- | --- |
| Up | 43 | NPPB, SPP1, IGFBP2, CHGB, DIRAS3Z, COMP, COLQ，CRLF1, VASH1，MXRA5, LTBP2, FRZB, COL21A1，SERPINE1，HSPA2, ANGPT2，RPL3L，THBS4，ENO2，DDAH1, COL3A1, TMEM159，NAV2，PPID，TNC, TNNT1, ANGPTL2，PXDN，INHBB，HK2, RGCC,CEMIP，DPYSL4，COL1A1, COPG1, COL5A1，KCNJ2，F2R, COL4A1，NDUFA4L2, MTCL1，FABP3，COL4A2, |
| Down | 105 | TGFBR2，MRC1，FGF12，IL6ST, TPR，DDX17，DCN, ALCAM, AKAP12, COLGALT2, SASH1，SEMA3C，SYNE2, LRRN3, MEIS3P1, FKBP5，EPHA4，HOPX，ZNF292，EPB41L2, EDNRA，GATB，CHRDL1，IGFBP6，NFKBIA，FGL2，CACNA2D2，VEZF1，SLC25A36，GAS1, SET，BTG2，ADD3, FGF7，OGN，TGFBR3，ANGPT1，KCTD12, SLC7A11，NR2F1，FAM129A, GSE1，BBX，C7，LXN，GPM6B，CDO1，NRIP1，TNPO1，NR1D，SLN，STAG2，CREB1，MT1X, RNF115，YAP1，ART3，CXADR，GSN，PTDSS1DSC1，NTRK2，TAGLN，MARCO，SEC22B，SOSTDC1, AR，CCL8，G0S2，CTSZ，ZNF148，RARRES1，USP13，RBP1，NT5DC2，PIK3R1，SELENBP1, MTUS2, ADH1B，ABCA8，TIMP3，PCYOX1，HLF，PIK3CA，MFAP4，COG5，CLU，CFD，PHACTR2，PLPP3，CHL1，C6，MYLK，DDR2，FMO2，SLIT2，PTGS2，PLP1，ALDH1A1，PPP1R1A，CYBRD1，SFRP5, ASTN2, BMP10, TNNI1 |
