# Supplementary material for: Involvement of plasminogen activator inhibitor-1 and its related molecules in atrial fibrosis in patients with atrial fibrillation
Source: PeerJ. 2021 Jun 2;9:e11488. doi: 10.7717/peerj.11488 (PMC8179226; doi:10.7717/peerj.11488)
Supplement: Supplemental Information 5 — N, number of patients; EF, ejection fraction; CCB, calcium channel blockers; SR, sinus rhythm; AF, atrial fibrillation. *P ¡ .05 vs. SR, **P ¡ .01 vs. SR. Values are presented as mean±SEM. Student’s t-test or Mann–Whitney U-test was used to evaluate differences between two groups. [file peerj-09-11488-s005.docx]

Supplementary Table3

Baseline characteristics of patients.

|  | SR | AF |
| --- | --- | --- |
| N | 64 | 113 |
| men(n) | 28 | 69 |
| women(n) | 36 | 44 |
| EF (%) | 65.59±4.34 | 64.33±11.20 |
| Diuretics (n) | 10 | 13 |
| Beta Blocker (n) | 3 | 6 |
| CCB (n) | 3 | 5 |

N, number of patients; EF, ejection fraction; CCB, calcium channel blockers; SR, sinus rhythm; AF, atrial fibrillation. ^⁎^P < .05 vs. SR, ^⁎⁎^P < .01 vs. SR. Values are presented as mean±SEM. Student's t-test or Mann–Whitney U‐test was used to evaluate differences between two groups.
