# Supplementary material for: Involvement of plasminogen activator inhibitor-1 and its related molecules in atrial fibrosis in patients with atrial fibrillation
Source: PeerJ. 2021 Jun 2;9:e11488. doi: 10.7717/peerj.11488 (PMC8179226; doi:10.7717/peerj.11488)
Supplement: Supplemental Information 7 — Raw data exported from the Cytoscape software applied for data analyses and preparation for Figs. 3D–3E. [file peerj-09-11488-s007.docx]

| **Cluster** | **Score (Density*#Nodes)** | **Nodes** | **Edges** | **Node IDs** |
| --- | --- | --- | --- | --- |
| 1 | 8.4 | 11 | 42 | COL1A1, COL3A1, COLGALT2,  COL4A2, SERPINE1, COL4A1,  COL21A1, COL5A1, DDR2, TIMP3, DCN |
| 2 | 4 | 13 | 24 | TGFBR2, GSN, IL6ST, PTGS2, CHRDL1, PIK3CA, YAP1, PIK3R1, TNC, CHGB, EDNRA, SPP1, F2R |
| 3 | 3.333 | 4 | 5 | NPPB, FABP3, TNNI1, TNNT1 |
| 4 | 3 | 3 | 3 | ANGPT2, ANGPT1, NTRK2 |
| 5 | 3 | 3 | 3 | HSPA2, PPID, FKBP5 |
| 6 | 3 | 3 | 3 | SLIT2, EPHA4, SEMA3C |
