# Supplementary material for: Involvement of plasminogen activator inhibitor-1 and its related molecules in atrial fibrosis in patients with atrial fibrillation
Source: PeerJ. 2021 Jun 2;9:e11488. doi: 10.7717/peerj.11488 (PMC8179226; doi:10.7717/peerj.11488)
Supplement: Supplemental Information 9 [file peerj-09-11488-s009.zip › Original western blots/Original western blots.pptx]

## Slide 1
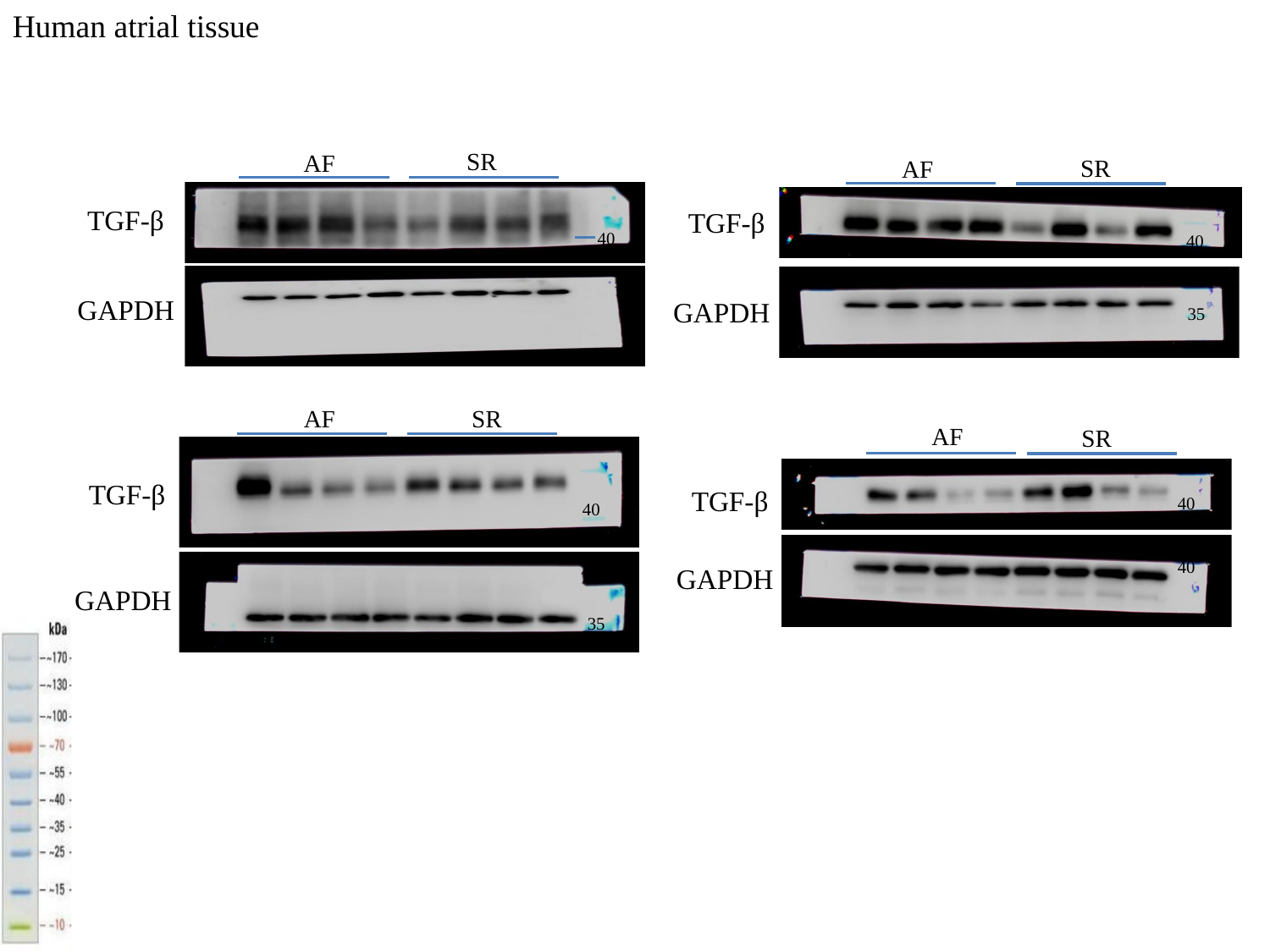

Human atrial tissue
SR
AF
SR
AF
TGF-β
TGF-β
40
40
GAPDH
GAPDH
35
AF
SR
AF
SR
TGF-β
TGF-β
40
40
40
GAPDH
GAPDH
35

## Slide 2
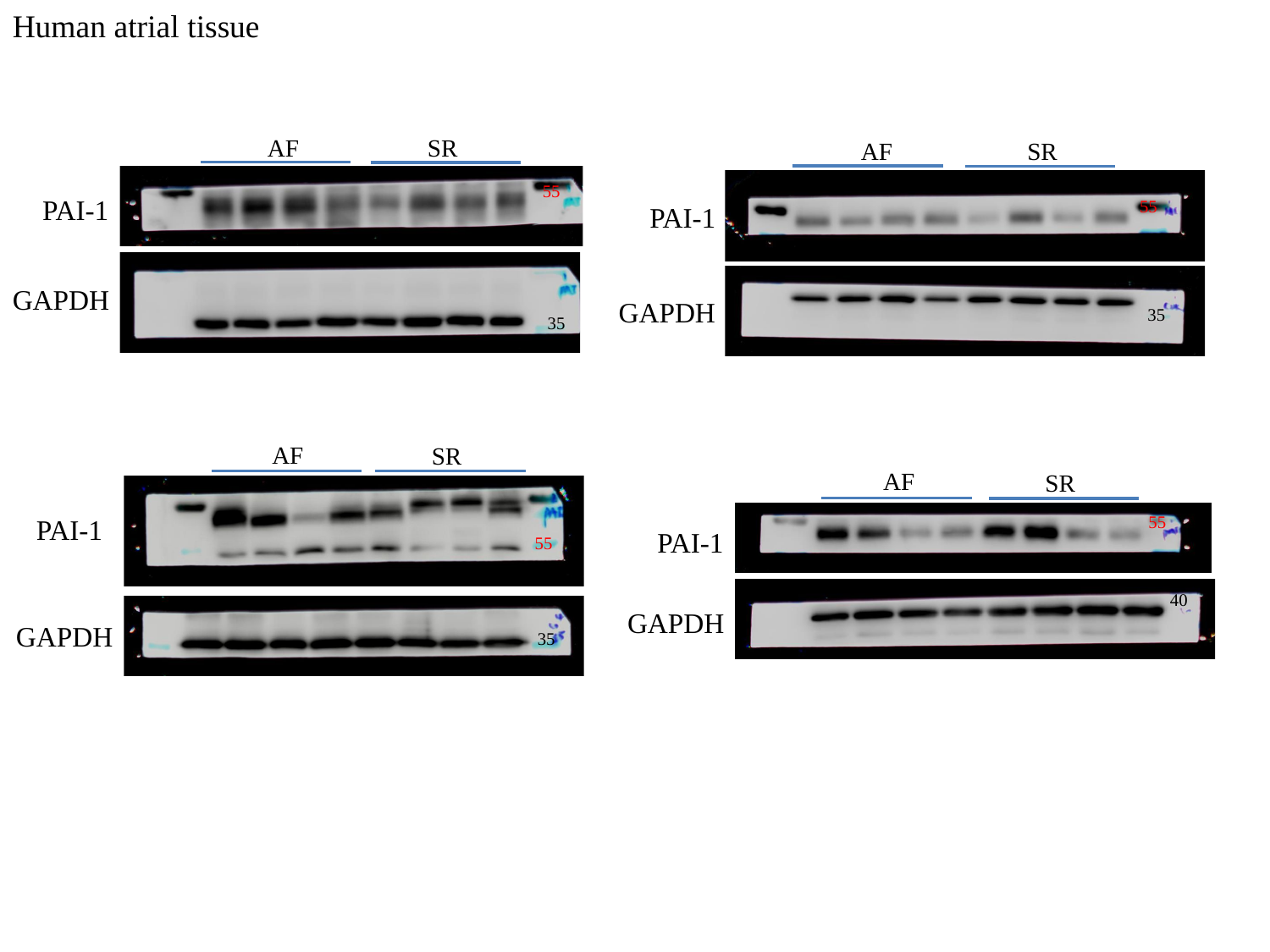

Human atrial tissue
AF
SR
AF
SR
55
PAI-1
55
PAI-1
GAPDH
GAPDH
35
35
AF
SR
AF
SR
55
PAI-1
PAI-1
55
40
GAPDH
GAPDH
35

## Slide 3
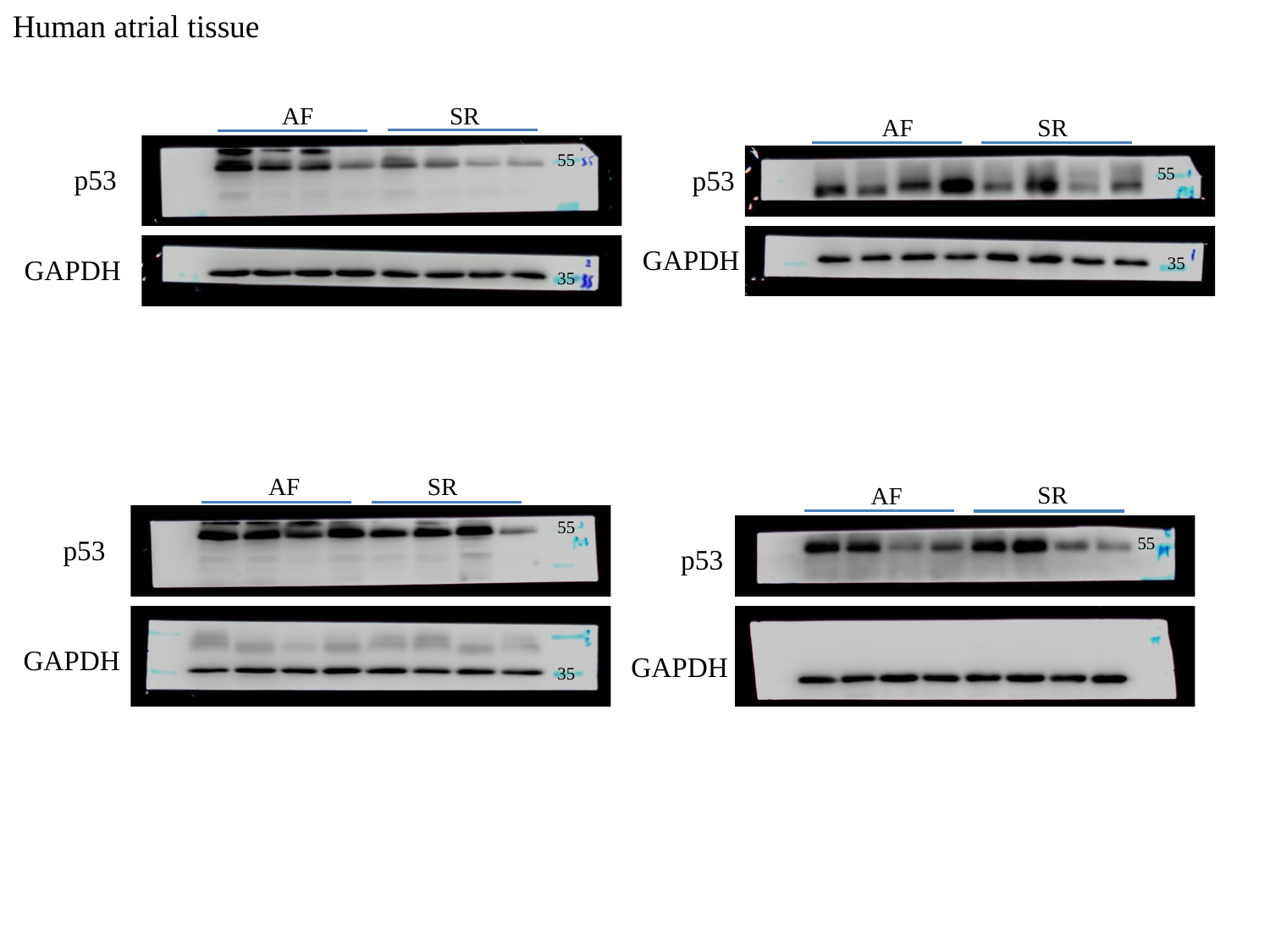

Human atrial tissue
SR
AF
AF
SR
55
p53
p53
55
GAPDH
GAPDH
35
35
AF
SR
SR
AF
55
p53
55
p53
GAPDH
GAPDH
35

## Slide 4
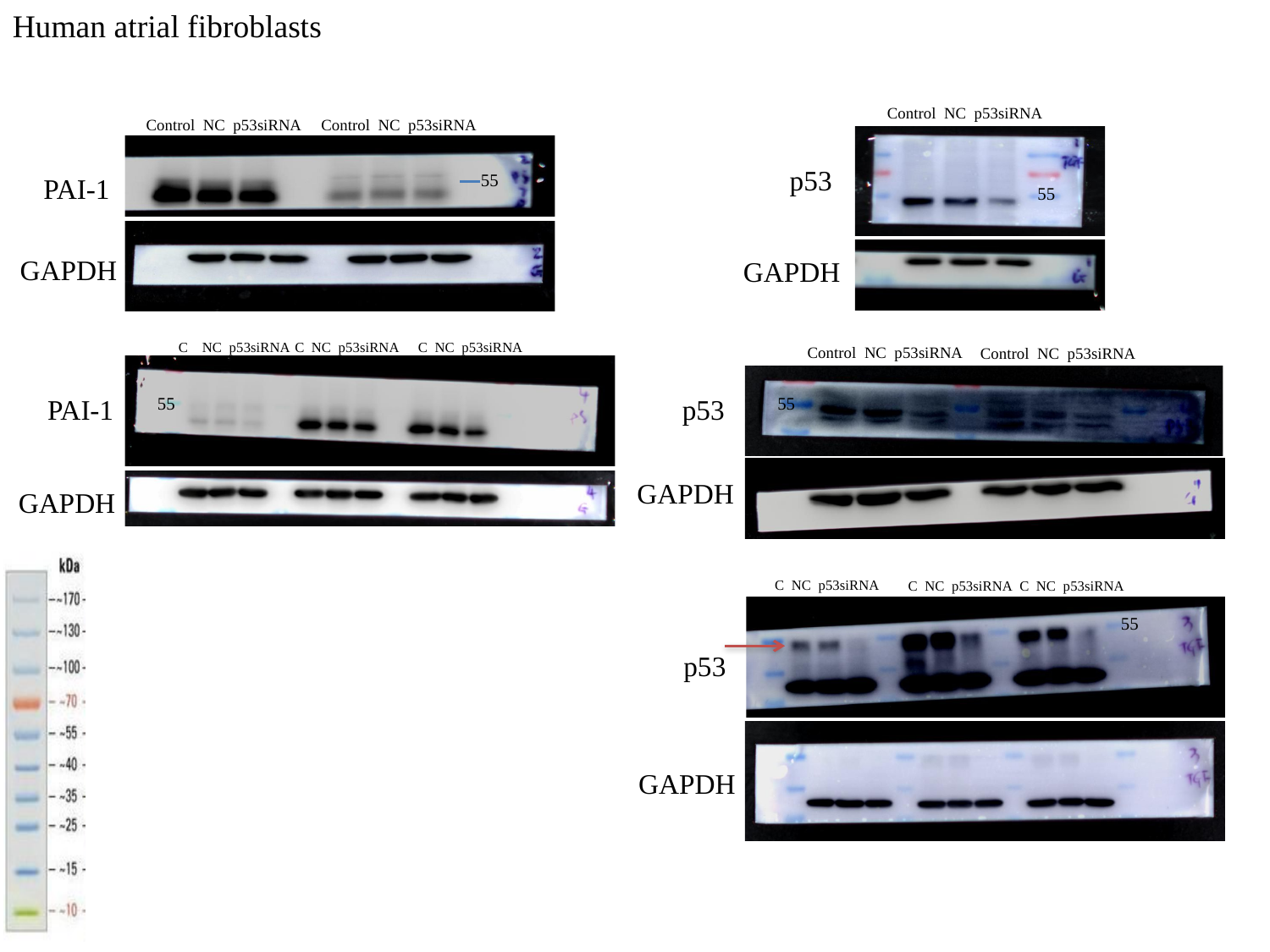

Human atrial fibroblasts
Control NC p53siRNA
Control NC p53siRNA
Control NC p53siRNA
p53
55
PAI-1
55
GAPDH
GAPDH
C NC p53siRNA
C NC p53siRNA
C NC p53siRNA
Control NC p53siRNA
Control NC p53siRNA
PAI-1
55
p53
55
GAPDH
GAPDH
C NC p53siRNA
C NC p53siRNA
C NC p53siRNA
55
p53
GAPDH

## Slide 5
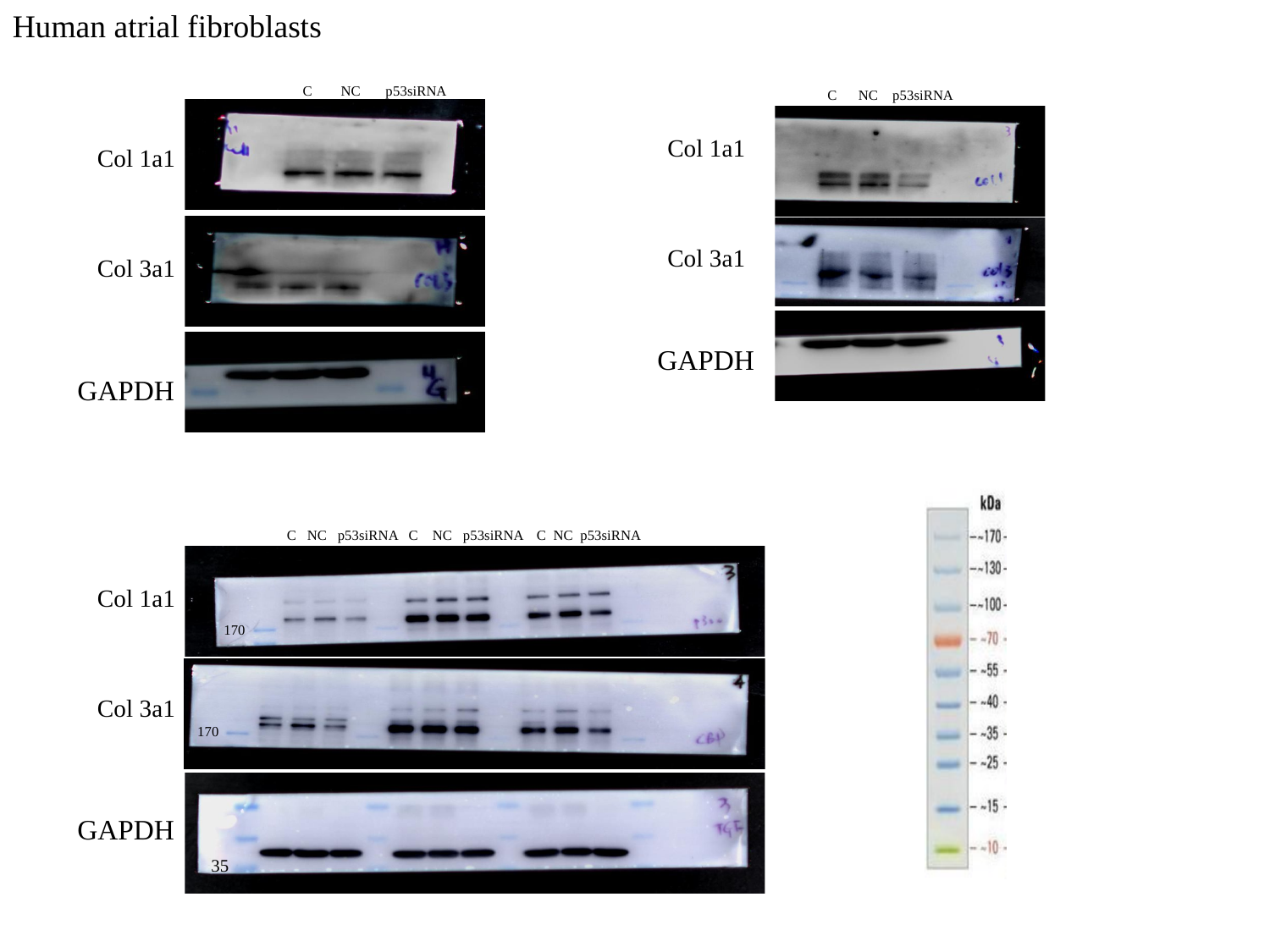

Human atrial fibroblasts
 C NC p53siRNA
C NC p53siRNA
Col 1a1
Col 1a1
Col 3a1
Col 3a1
GAPDH
GAPDH
 C NC p53siRNA
C NC p53siRNA
C NC p53siRNA
Col 1a1
170
Col 3a1
170
GAPDH
35
